# Ring Expansion of Vinylaziridines through the Strain-Release Pericyclic Reaction: Recent Developments and Applications

**DOI:** 10.3390/molecules18089650

**Published:** 2013-08-12

**Authors:** Yu Mi Heo, Seung-Mann Paek

**Affiliations:** College of Pharmacy and Research Institute of Pharmaceutical Sciences, Gyeongsang National University, Jinju daero, Jinju, Gyeongnam 660-751, Korea; E-Mail: ymh@gnu.ac.kr

**Keywords:** vinylaziridines, cycloaddition, rearrangement, ring expansion

## Abstract

Recent syntheses of azetidines, pyrrolidines, piperidines and azepines through cycloaddition or sigmatropic rearrangements of vinylaziridines are described. Applications to natural product synthesis and mechanistic investigations are also summarized.

## 1. Introduction

**Scheme 1:**
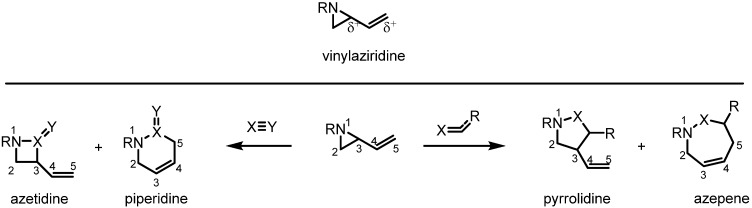
Synthetic applications of the vinylaziridine moiety.

Vinylaziridines, which simultaneously possesses electrophilic and nucleophilic centers in their structure, have been regarded as highly valuable synthetic intermediates because of their unique reactivity toward various other reactive species such as activated alkenes, heteroatoms and metallic species [1]. Since the expansion of a vinylaziridine via sigmatropic rearrangement was first ring reported in 1967 [2,3], a variety of these transformations have been shown to offer efficient and valuable synthetic routes to a wide variety of products. Nowadays the vinylaziridine moiety plays an important role in the preparation of 4-, 5-, 6- and 7-membered azacycles according to the selection of reagents and reaction conditions (Scheme 1). Because this transformation releases the severe ring strain of the aziridine ring, this conversion usually proceeds in a highly efficient manner. In light of this unique reactivity and effectiveness, a lot of synthetic research in this area is being carried out even now. In this review recent developments and application of these methodologies in the last decade are summarized.

## 2. Results and Discussion

### 2.1. Azetidines from Vinylaziridines

**Scheme 2:**
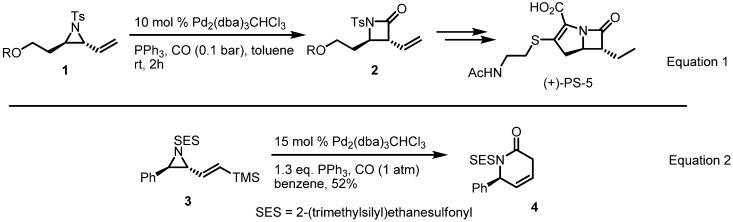
Synthesis of β and δ-lactams from the vinylaziridine.

Synthesis of a β-lactam from a vinylaziridine through insertion of carbon monoxide was published in 1993 (Equation 1 in Scheme 2) [4]. This enabled the preparation of the β-lactam antibiotic PS-5 and initiated studies into related transformations. In the early 2000s, however, a silyl-substituted vinylaziridine was converted into a 6-membered azacycle under similar reaction conditions (Equation 2 in Scheme 2) [5].

**Scheme 3:**
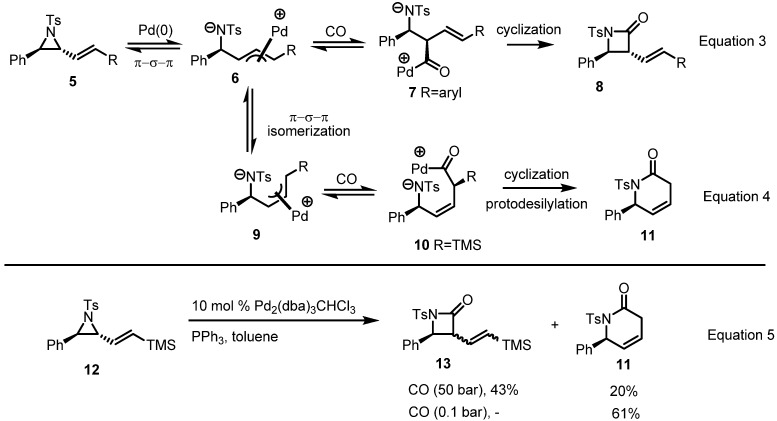
Mechanism of β and δ-lactam formation from vinylaziridines.

A recent report from the Aggarwal group at Bristol University suggests a plausible explanation of this discrepancy (Scheme 3) on the basis of a Pd-mediated isomerization and CO insertion mechanism [6]. Once CO is inserted into the carbon, the so-formed π-allyl complex **6** is dominantly and quickly converted to β-lactam **8** (Equation 3 in Scheme 3), but when the substituent is a silyl group, carbonylation occurs at the carbon adjacent to silicon because of the shorter C-Pd bond length [7]. Once Pd(0) is inserted into the carbon adjacent to silicon, it affords cyclization product **11** after protodesilylation (Equation 4 in Scheme 3). Based on this explanation and hypothesis, various reaction conditions were surveyed to control the regioselectivity of this conversion, as shown in Equation 5 in Scheme 3.

### 2.2. Pyrrolidines from Vinylaziridines

**Scheme 4:**
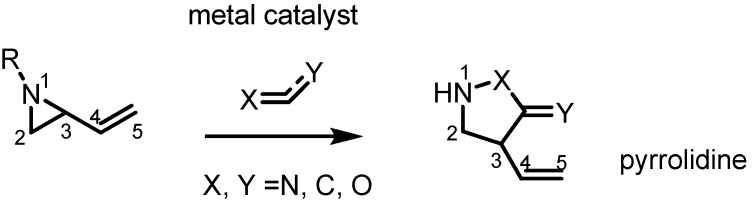
Synthesis of 5-membered azacycles from vinylaziridines.

Most recent pyrrolidine syntheses from vinylaziridines apply transition metal catalysts, especially Pd(0) species, with an activated π-bond as shown in Scheme 4. In the presence of the metal catalyst the nucleophilic nitrogen and electrophilic C3 cyclize with the activated π-bond in a stereoselective manner.

**Scheme 5:**
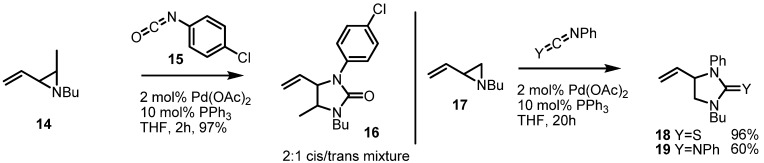
Synthesis of 5-membered cyclic ureas from vinylaziridines.

For example, the Alper group reported in 2000 a highly efficient synthetic protocol for preparing 5-membered urea skeletons **16** using Pd(OAc)_2_, PPh_3_ and aryl isocyanates (Scheme 5). Instead of isocyanate, carbodiimide or thioisocyanate could also be used to give the corresponding azacycles **18** or **19**, respectively in good yield [8]. Because this versatile transformation needed phosphine ligands for the catalytic cycle, it offered the possibility of using chiral ligands to control the enantioselectivity of these reactions.

**Scheme 6:**
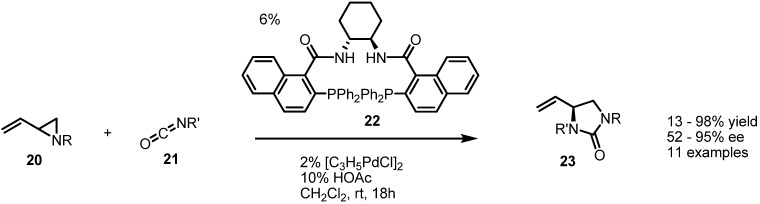
Enantioselective synthesis of cyclic ureas from vinylaziridines.

In fact, the Trost group has published a chiral version of this transformation employing the Trost ligand **22** (Scheme 6) [9]. Cycloaddition of benzyl, vinylaziridine **20** and benzyl isocyanate **21** with catalysis by Pd(0) and a chiral phosphine ligand produced the chiral cyclic urea **23** in a highly efficient and enantioselective manner.

This reaction could be applied to other aromatic or benzylic aziridines and isocyanates with similar results. This result is a good example of how strain-release cycloaddition can be applied to enantioselective catalysis.

**Scheme 7:**
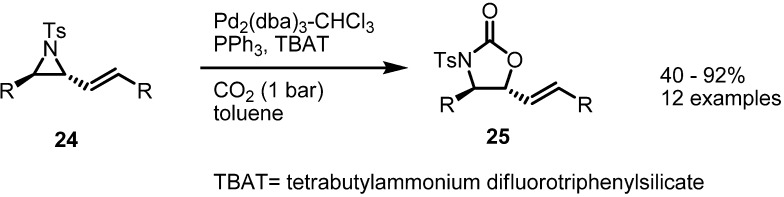
Formation of oxazolidinones from vinylaziridines.

Instead of isocyanate, CO_2_ could be utilized as a π-bond partner. The Aggarwal group reported an application of this transformation with CO_2_ to form substituted oxazolidinones [10] (Scheme 7). When aryl vinylaziridine **24** was treated with Pd(0), PPh_3_, CO_2_ and a quaternary ammonium salt, the desired oxazolidinone **25** was obtained in high yield. This transformation could be performed with various substrates without loss of chirality. Noticeable is the fact that simple CO_2_, although nonpolar, could be incorporated in this bond formation. The electrophilicity of the CO_2_ carbon atom is thought to play an important role in the reaction outcome.

**Scheme 8:**
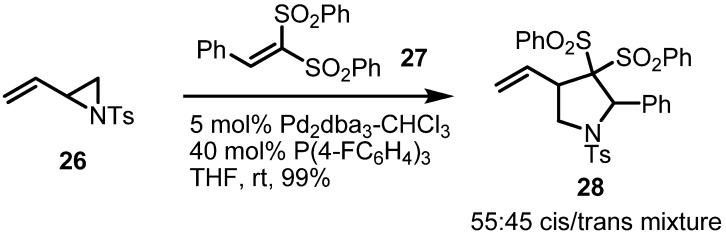
Formation of pyrrolidines from a vinylaziridine and an activated alkene.

An alkene can be used in this transformation as a π-bond partner if it is activated with other substituents. The Yamamoto group demosntrated this type of conversion in 2002 [11]. As shown in Scheme 8, trisubstituted alkene **27** is highly electron deficient because of its two sulfonyl groups. This highly activated π-bond reacted with simple vinylaziridine to produce pyrrolidine **28** in almost quantitative yield, although with low stereoselectivity. This reaction could be applied to other similar substrates in over 69% yield.

**Scheme 9:**
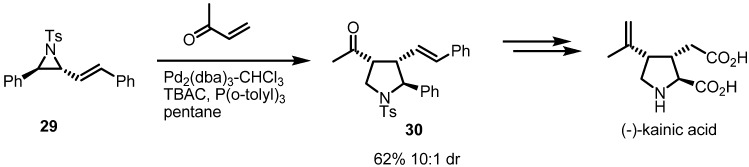
Total synthesis of (−)-kainic acid.

Improvement for this type of reaction was recently achieved by the Aggarwal group [12]. To expand the synthetic applications, methyl vinyl ketone (MVK) was utilized as a Michael acceptor. This replacement of disulfonyl alkene **27** with MVK afforded a general stereoselective route to the pyrrolidine skeleton. This transformation wase utilized successfully in a total synthesis of kainic acid (Scheme 9). This beautiful example shows that the strain-release cycloaddition of vinylaziridines can be applied to synthesize complex natural products.

**Scheme 10:**
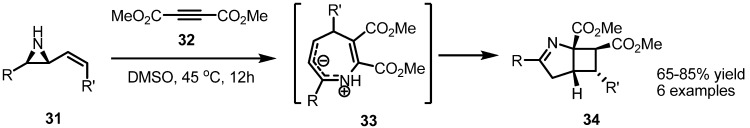
Preparation of bicyclic pyrrolines.

Like alkenes, electron deficient alkynes also can be utilized in this cycloaddition. Thus the highly electron deficient alkyne **32** was applied to obtain the highly strained [3.2.0] bicyclic diester **34** [13] (Scheme 10). This transformation could be carried out without any metal catalyst because of the intrinsic electrophilicity of alkyne **32**, like in the case of CO_2_. The seven-membered azacycle **33** was suggested as a plausible intermediate.

**Scheme 11:**
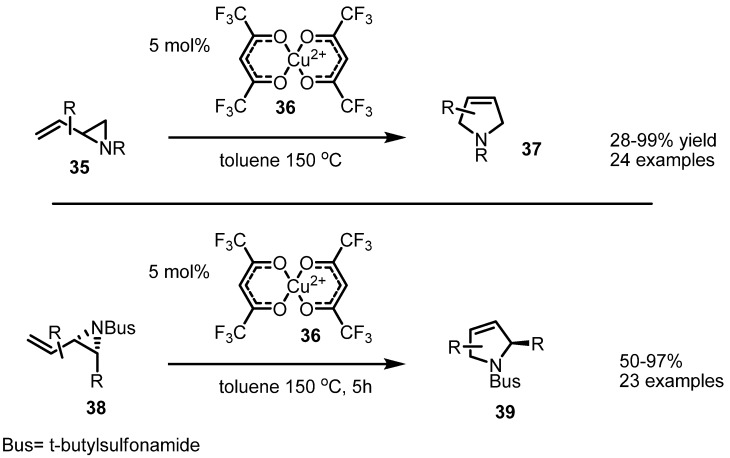
Copper catalysis of a [1.3]-rearrangement.

Instead of Pd (0) species, a copper catalyst was found to be useful in an isomerization of vinylaziridines. Njardarson and coworkers reported that the synthesis of substituted pyrrolidines **37**, **39** can be achieved with copper species catalysis and anhydrous hexafluoroacetylacetonate ligand [14]. This reaction is expected to proceed via a 1,3-rearrangement mechanism [15]. This conversion is also applicable to variously substituted vinylaziridines, as shown in Scheme 11 [16].

**Scheme 12:**
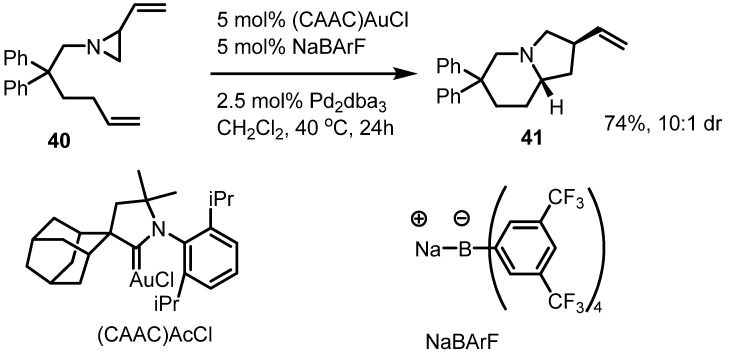
Dual metal catalysis of vinylaziridines.

Dual metal catalyzed ring enlargement of vinylaziridines was reported in 2012. The Blum group reported that Au/Pd dual catalysis of substituted vinylaziridine **40** afforded the pyrrolo[1,2a]pyridine **41** in good yield and diastereoselectivity [17] (Scheme 12). Although the complex catalyst and ligand structure hamper its wide application, this result shows that the combination of metal catalysts and ligands can be optimized in this valuable transformation.

**Scheme 13:**
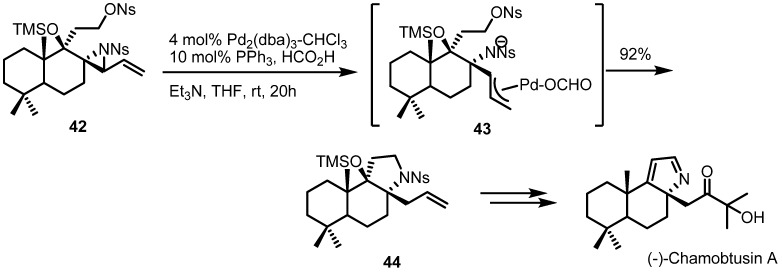
Total synthesis of (−)-chamobtusin A.

A Pd-catalyzed ring expansion reaction was also utilized recently in the total synthesis of the natural product (−)-chamobtusin A [18] (Scheme 13). The Aoyagi group reported that a Pd(0)-mediated ring opening of vinylaziridine/C-N bond formation/reductive elimination sequence afforded the tricyclic alkaloid **44** in 92% yield. This synthetic intermediate was then utilized for the total synthesis of (−)-chamobtusin A. This result is another good example of the synthetic efficiency of the strain-release reactions of vinylaziridines.

**Scheme 14:**
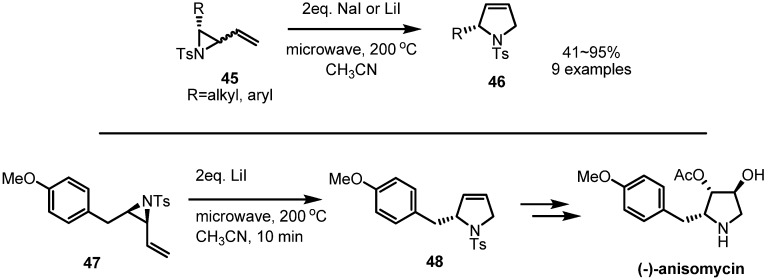
Thermal rearrangement of a vinylaziridine and its application to the synthesis of (−)-anisomycin.

Without metal catalysis, thermal rearrangements of vinylaziridines were also reported [19]. Once the vinylaziridines **45** were heated under microwave, the corresponding pyrrolidine derivatives **46** were produced in high yield, as shown in Scheme 14. Employing this procedure, a formal synthesis of (−)-anisomycin, a natural antibiotic, could be performed. Thermal activation of the substituted vinylaziridine **47** afforded the desired PMB substituted pyrrolidine **48** which could be transformed into (−)-anisomycin by known procedures [20].

**Scheme 15:**
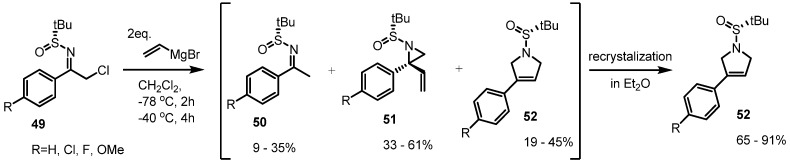
Spontaneous rearrangement of vinylaziridines to pyrrolidines.

More spontaneous rearrangements of vinylaziridine were reported in 2011 [21]. During treatment with vinylmagnesium bromide, the desired transformations from the sulfinylketimines **49** to the corresponding vinylaziridines **51** were observed in the crude reaction mixture (Scheme 15). The ^1^H-HMR analysis showed formation of the desired vinylaziridine derivatives **51** in 33%–61%, 9%–35% of dehalogenated ketimines **50** and 19%–45% of the pyrrolidine derivatives **52** altogether. After purification of the reaction mixture, however, the rearranged pyrrolidine derivatives **52** were isolated as the exclusive major products. This observation means that spontaneous ring expansions of vinylaziridines can also be performed, depending on their substitution pattern.

### 2.3. Piperidines from Vinylaziridines

**Scheme 16:**
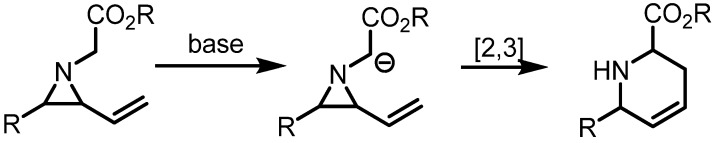
Preparation of pyridine derivatives through [2,3]-sigmatropic rearrangement.

Since the reports of the Coldman group and Somfai group respectively, the [2,3]-rearrangement of vinylaziridines has been regarded as an efficient methodology to prepare 6-membered azacycles [22,23] (Scheme 16).

**Scheme 17:**
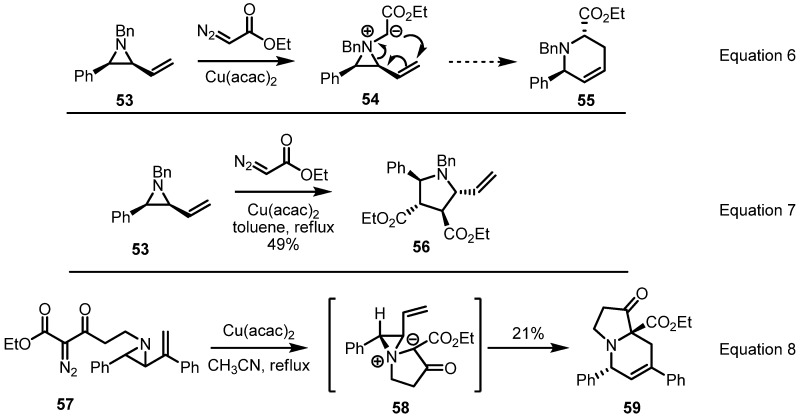
Preparation of pyridine derivatives through [2,3]-sigmatropic rearrangement.

Based on the previous result, modification of this conversion was reported in 2004 by the Rowlands group. They had planned that a carbene insertion of diazoacetate into the aziridine would produce an aziridinium ylide **54** to afford the [2,3]-Stevens rearrangement product **55** stereoselectively (Equation 6 in Scheme 17) [24]. After extensive survey of reaction conditions, however, this conversion was shown to be hampered by various side reactions or dimerization of metal carbenoid (Equation 7). To avoid this side reaction, instead of intermolecular carbene insertion, intramolecular insertion as shown in Equation 8 was used. When a copper catalyst was added and the reaction mixtures heated in acetonitrile, a [3,5] fused zwitterionic intermediate **58** was formed and converted to desired pyrrolo[1,2a]pyridine derivative **59** in low yield. Although this low chemical yield still represents a drawback of this transformation, the well-designed reaction plan and complex framework of the product are expected to be utilized for more developments and applications.

### 2.4. Azepines from Vinylaziridines

**Scheme 18:**
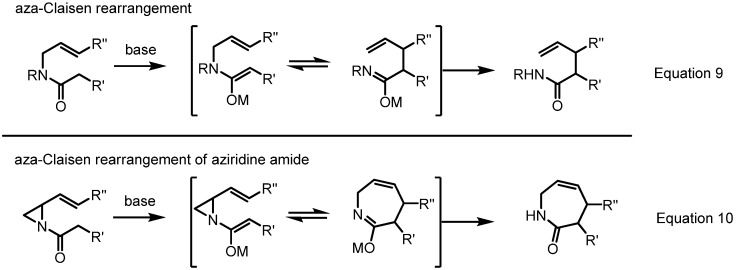
[3,3]-Sigmatropic rearrangement of vinylaziridines.

Just as a [2,3]-rearrangement of vinylaziridines produces 6-membered azacycles, a [3,3]-rearrangement affords 7-membered azacycles, *i.e.*, azepine skeletons (Scheme 18). It should be noted that this type of ring expansion for vinyl aziridine was historically the first such reaction to be reported [2,3] (Equations 9 and 10 in Scheme 18). So far, many variations or applications of this conversion have been published because it can readily produce generally otherwise unavailable azepine skeletons in a stereoselective manner.

**Scheme 19:**
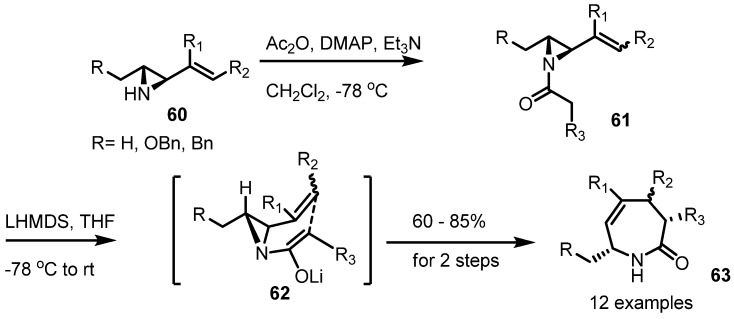
Aza-Claisen rearrangement of vinylaziridines.

For example, the Somfai group showed a successful synthesis of the substituted azepine **63** using this rearrangement [25]. Because this transformation is a strain-release reaction, the product **63** is thought to be kinetically favored (Scheme 19).

**Scheme 20:**
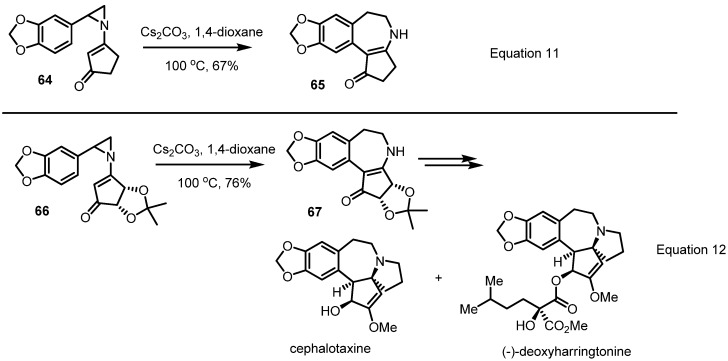
Total synthesis of cephalotaxine and deoxyharringtonine.

More dramatic application of this conversion to natural product synthesis was achieved by the late Prof. Gin in 2007 [26]. For a total synthesis of cephalotaxine and related natural products, this sigmatropic rearrangement of the substituted vinylaziridine **64** was carried out and found to produce the desired benzazepine **65** in moderate yield with just the aid of cesium carbonate in 1,4-dioxane solution (Equation 11 in Scheme 20). Employing this protocol, application to the chirally substituted vinylaziridine **66** was examined. As expected, smooth conversion to the desired benzazepine **67** without loss of chirality was observed. With this efficient skeleton preparation method, a successful synthesis of cephalotaxine and the related natural product (−)-deoxyharringtonine, a potent a nti-leukemia alkaloid, could be completed (Equation 12 in Scheme 20).

**Scheme 21:**
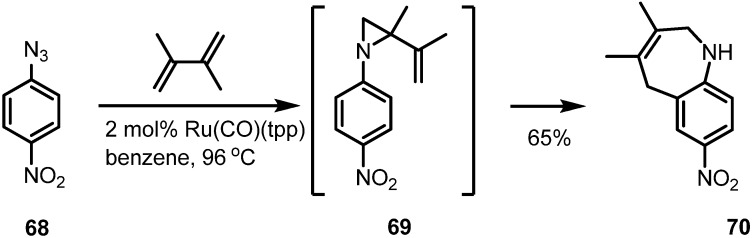
Ruthenium catalyzed aza-Claisen rearrangement.

A metal catalyzed version of this type of transformation has also been studied. The Gallo group reported a similar conversion using a ruthenium catalyst as shown in Scheme 21 [27].

Thermal activation of aromatic azide **68** produced an aryl-nitrene to afford a vinylaziridine **69** via nitrene insertion into the alkene moiety. This corresponding vinylaziridine was transformed into the desired benzazepine product **70** in good yield.

**Scheme 22:**
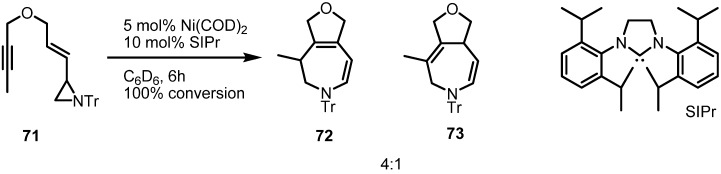
NHC-assisted rearrangement of the vinylaziridine **71**.

Nickel catalyzed rearrangement of the aziridinylenynes **71** could also be performed with the* N*-heterocyclic carbene ligand, SIPr [28] (Scheme 22). Ether-tethered vinylaziridine and alkyne cyclize to a mixture of furo[3,4d]azepines **72** and **73** in good conversion. It is interesting that phosphine ligands gave no conversion of the substrate. Although this reaction still needs more development in terms of chemical yield and substrate scope, it has room for application of chiral catalysts and ligands.

**Scheme 23:**
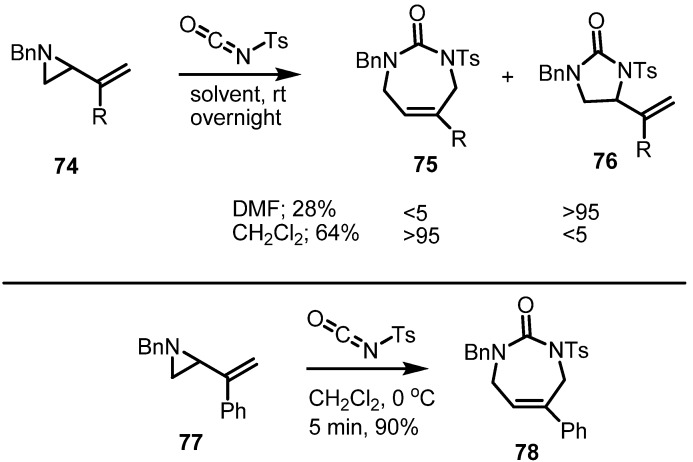
Cycloaddition of isocyanates to form azepine derivatives.

Independently of the sigmatropic rearrangement, a cycloaddition of isocyanates with vinylaziridines was also executed. When tosyl isocyanate was added to the benzyl vinylaziridine **74**, the azepine **75** was obtained in good yield (Scheme 23). It should be noted that choice of solvent can influence the regioselectivity of this conversion [29]. Thus, with DMF as solvent, imidazole derivative **76** was produced as the major isomer, albeit in low chemical yield. When DMF was replaced by CH_2_Cl_2_ as solvent, the desired azepine derivatives were produced in good chemical yield and regioselectivity. After an extensive optimization of the reaction conditions, the vinylaziridine **77** could be converted into the urea **78** in 90% yield.

## 3. Conclusions

Recent advances in the strain-release ring enlargement of the vinylaziridine moiety were described. Owing to their unique reactivity, vinylaziridines are still regarded as attractive synthetic intermediates, as summarized above. Their further variations, improvements and applications will be a highly active research topic because the reaction still has unmet needs in terms of regio- and stereoselectivity. With this synthetic endeavor, it can be expected that more fruitful results will be realized, just as they were in the last decade.
